# Floating novel object recognition in adult zebrafish: a pilot study

**DOI:** 10.1007/s10339-019-00910-5

**Published:** 2019-02-27

**Authors:** István Magyary

**Affiliations:** University of Pécs, KPVK, Rákóczi u. 1, 7100 Szekszárd, Hungary

**Keywords:** Adult zebrafish, Novel object recognition, NOR, Floating objects

## Abstract

The novel object recognition (NOR) tasks can be used to quantify memory function in zebrafish similarly to rodents. The development of zebrafish learning and memory tests provides a means for testing the effects of pharmacological manipulations of memory. Several authors reported on the successful application of different objects in NOR tests placed either at the bottom of test tanks or submerged into the tank water of zebrafish. This pilot study was designed to test the suitability of floating objects in NOR tests using adult zebrafish. Floating objects such as crumpled aluminum balls and pink plastic hollow pearls were found to be suitable for NOR tests when small groups of zebrafish are used as experimental animals. Adult zebrafish of both sexes were capable of distinguishing between the different colors and surface consistencies of certain floating objects. A significantly higher number of mouth-object contacts were recorded when either floating aluminum balls or floating plastic pearls were used as novel object during NOR tests.

## Introduction

Zebrafish feed chiefly in the water column, and their diet consists mainly of zooplankton and insects. However, terrestrial insects are also consumed, suggesting surface feeding (Spence et al. 2008).

In the laboratory environment, anxious fish dwell initially at the bottom of a novel tank and frequently exhibit freezing behavior and a generalized reluctance to approach the water surface. Zebrafish come up to the surface for food in situations when their innate sense of risk for danger and predation are minimal and the desire for nourishment surpasses that fear (De Lombaert et al. 2017).

The novel object recognition tasks can be used to quantify memory function in zebrafish similarly to rodents. At first, the zebrafish is allowed to explore two identical objects, e.g., small plastic figures or cubes. After this exploration condition, the zebrafish is presented with the previously encountered object and a novel object. Time spent exploring the novel object, over the familiar one, is characterized as an index of memory (Antunes and Biala 2012).

The development of zebrafish learning and memory tests provides a means for testing the effects of pharmacological manipulations of memory (May et al. 2016) such as the effects of scopolamine (Hamilton et al. 2017). Several authors reported on the successful application of different stationary objects in NOR tests placed either at the bottom of test tanks or submerged into the tank water of zebrafish (e.g., Hamilton et al. 2017; Lucon-Xiccato and Dadda 2014) or presented as virtual objects by side walls (Braida et al. 2014).

This pilot study was designed to test the suitability of floating non-stationary objects in novel object recognition tests using adult zebrafish.

## Materials and methods

**Fig. 1:**
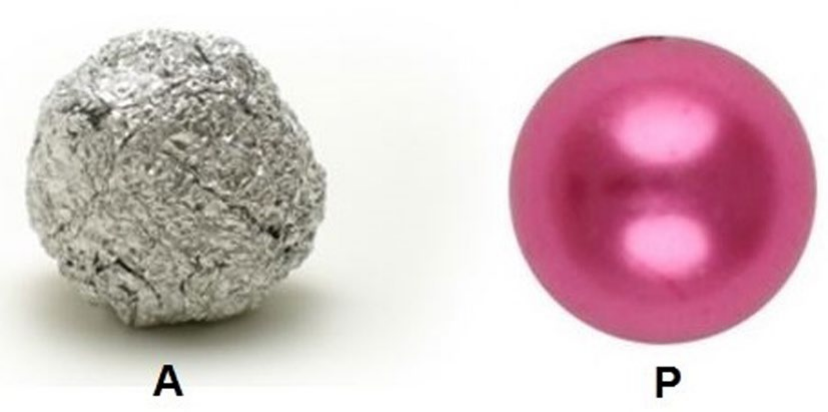
Test objects: crumpled aluminum foil balls (**A**), pink hollow plastic pearl (**P**)

**Fig. 2:**
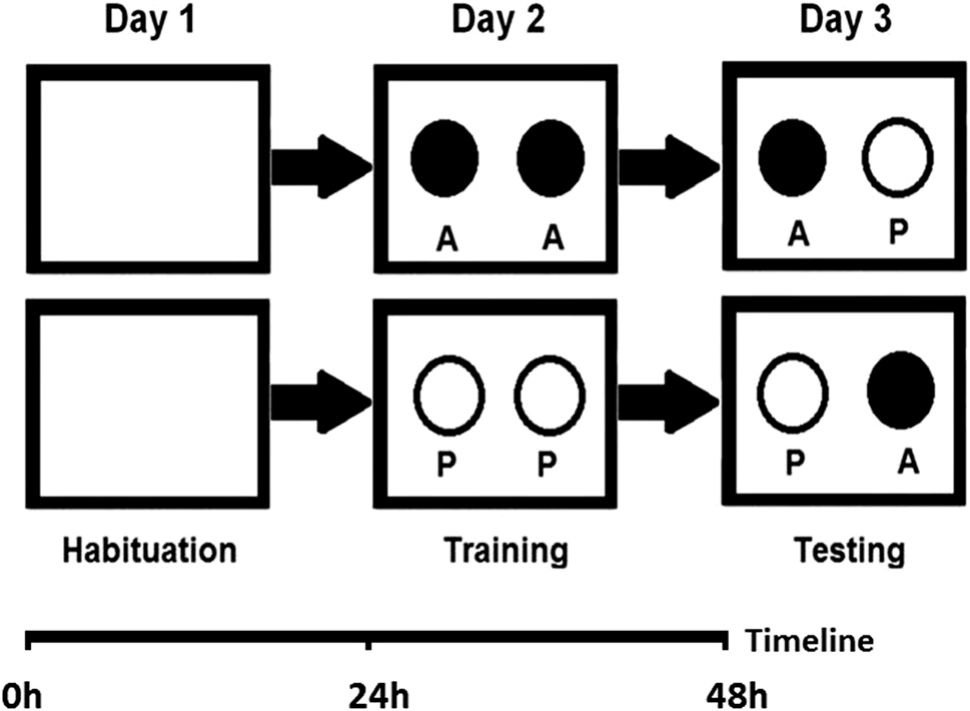
Experimental design: day 1: habituation, day 2: training, day 3: testing (using novel object)

Adult healthy wild-type commercial zebrafish of both sexes were used for experiments. (Strain was kindly provided by Dr. Ferenc Baska, University of Veterinary Medicine, Budapest, Hungary). Fish were kept in glass tanks of 100 L (stocking density: 2 fish/L) using commercial food for daily feeding and 14 h:10 h light–dark cycle prior to the experiments. Water temperature was maintained at 24 °C. For the analysis of NOR, groups of 4 zebrafish were examined (*n* = 10). The fish were kept and observed in opaque walled glass tanks (200 mm long, 150 mm wide and 100 mm high) in a water column of 50 mm. Experimental groups were kept under these conditions without feeding until completing the final tests on day 3 (see timeline on Fig. 2). In order to allow sufficient time for adaptation, experimental fish were transferred to test tanks 24 h prior to testing (day 1: habituation, Fig. 2). Test tanks were illuminated through the transparent bottom glass using homogenous white light. Fish were allowed to adapt to the illuminated tank for 15 min before behavioral recording was started. On day 2 either two crumpled aluminum foil balls (floating, 5 mm diameter each) or plastic pearls (floating, 6 mm diameter, pink) were placed at the same time into the test tank (Figs. 1, 2) for 10 min (training).

**Fig. 3:**
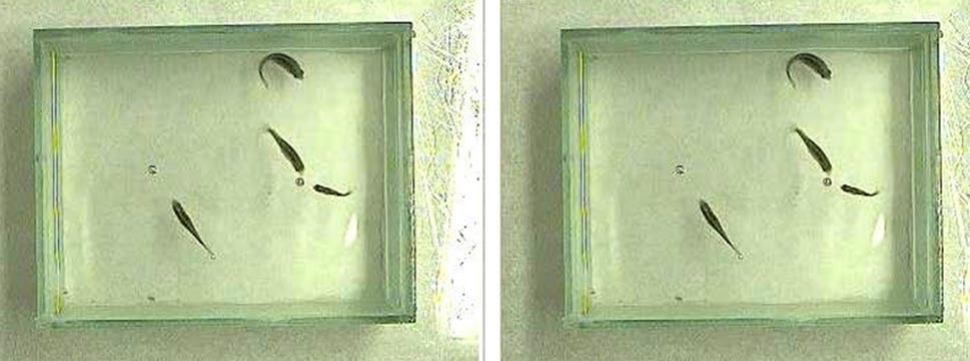
Mouth-object contacts/ball were counted in each frame of 10-min video captures

On day 3, one aluminum foil ball and one plastic pearl were placed at the same time into the test tank (Figs. 1, 2). Novel object recognition behavior of fish was recorded by using a KS 720 ½” CCD Color/B/W video camera (NET GmbH, Germany) in AVI format. Ten min was recorded in each experiment using 5 frames/s frequency. The recorded video data were processed by using the VirtualDub (1.7.6) software so that the number of mouth-object contacts was counted in corresponding frames (Fig. 3).

Experiments were carried out in duplicate, whereas mouth-object contacts/ball were counted in 10 groups of zebrafish in each experiment (*n* = 10/experiment).

### Statistical analysis

Student’s *t* tests were used to analyze the preference of tested zebrafish groups (cumulative data of 4 fish/group) for the different objects. The level of significance was taken as *p* < 0.05. Statistical analyses were done using software JASP, version 0.8.6.

## Results

**Fig. 4:**
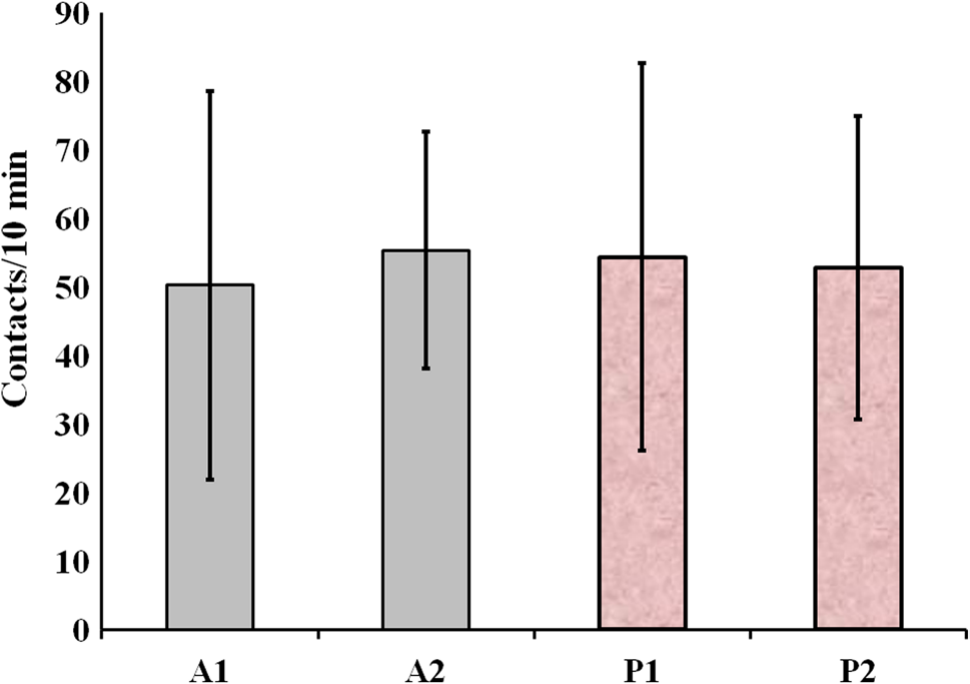
The numbers of mouth-object contacts are compared by each object (A1, A2, P1, P2) during training on day 2. No significant differences were found

No significant differences were found when the numbers of mouth-object contacts were compared either in the case of two aluminum balls (*A*1 and *A*2) or in the case of two plastic pearls (*P*1 and *P*2) during training on day 2 as outlined by a paired-samples *t* test (*A*1: *M* = 50.20, SD = 28.34; *A*2: *M* = 55.30, SD = 17.27), *t*(*df* = 9, *n* = 10) = −0.414, *p* = 0.689; (*P*1: *M* = 54.30, SD = 28.23; *P*2: *M* = 52.70, SD = 22.10), *t*(*df* = 9, *n* = 10) = 0.132, *p* = 0.898 (Fig. 4).

**Fig. 5:**
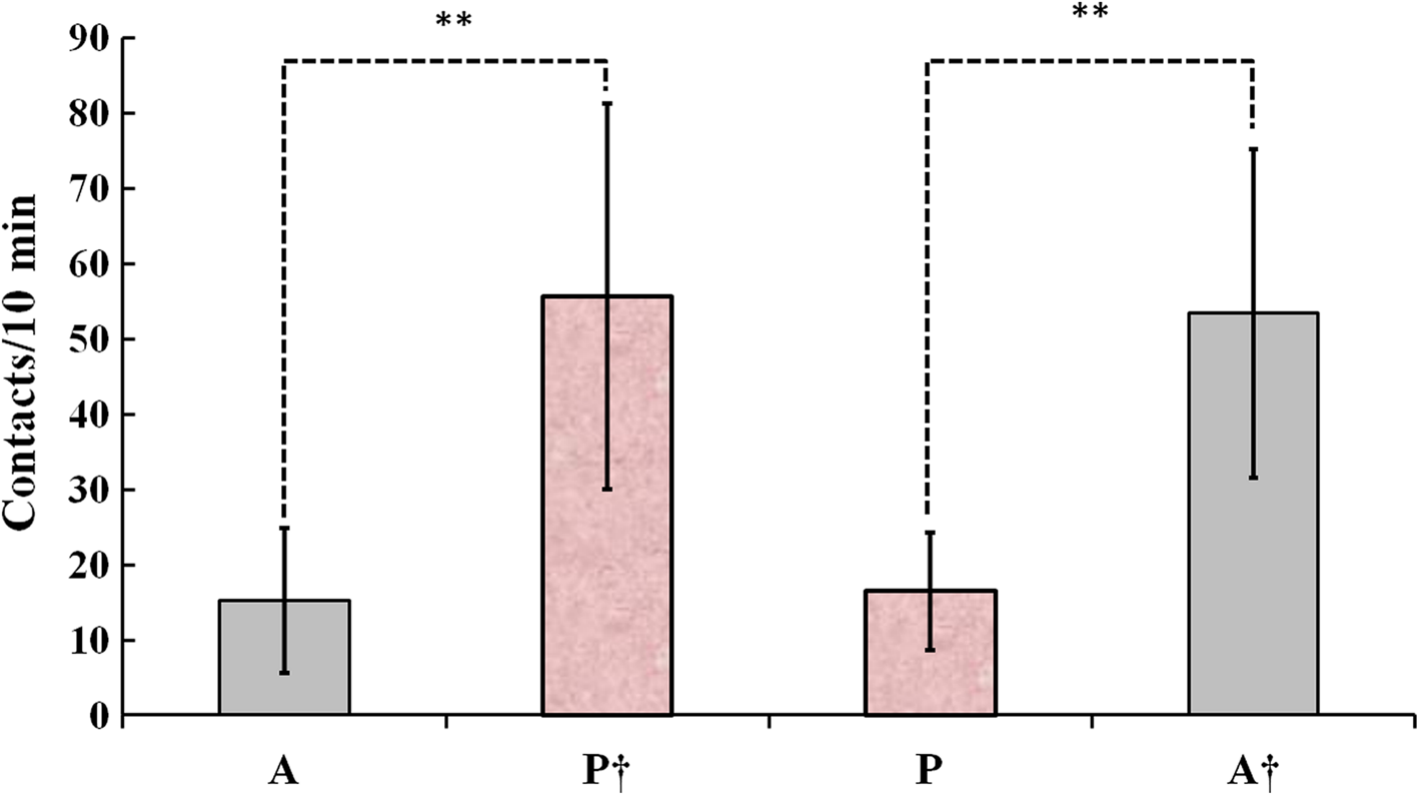
Both floating plastic pearl (P) and aluminum ball (A) induced significantly higher number of mouth-object contacts as novel object when tested 24 h after training. **p < 0.001, ^**†**^novel object, *n* = 10

A significantly higher number of mouth-object contacts were recorded when either floating plastic pearls (*P*^**†**^) or aluminum balls (*A*^**†**^) were used as novel object during testing as outlined by paired-samples *t* tests. *P*^**†**^: [*A*: *M* = 15.20, SD = 9.636; *P*: *M* = 55.60, SD = 25.648), *t*(*df* = 9, *n* = 10) = −5.034), *p* < 0.001]; *A*^**†**^: [*P*: *M* = 16.50, SD = 7.821; *A*: *M* = 53.40, SD = 21.829), *t*(*df* = 9, *n* = 10) = −6.102), *p* < 0.001] (Fig. 5).

Floating object type (*A* or *P*) had no significant effect on mouth-object contacts.

## Discussion

Zebrafish in their natural habitat are inhabiting slow-moving or standing water bodies, the edges of streams and ditches adjacent to rice-fields. In order to avoid predatory birds and other predators, zebrafish shoals preferably hide under floating plants and they retain this behavior under laboratory conditions, where both males and females prefer the tank compartment with floating plants over a barren one (Schroeder et al. 2014). The high rate of planktonic organisms in their diet indicates that zebrafish feed mainly in the water column. However, terrestrial insects are also consumed, suggesting surface feeding as described above. In a shoaling species such as zebrafish, fish perform better in groups and stress restricts learning ability in isolated individuals (Spence et al. 2008). In this pilot study, groups (shoals) of 4 adult zebrafish could successfully adapt to the relatively small volume of test tanks so that 24 h of habituation period (day 1) was sufficient, allowing training with two identical objects and testing the novel object recognition behavior on day 2 and day 3. The experimental design described hereby was similar to that of rodents where the NOR task procedure consists of the following three phases: habituation, familiarization and test phase (Antunes and Biala 2012). Zebrafish kept and tested in these conditions and within shoals could easily overcome anxiety of novel and barren tank and as being kept without feeding their desire for nourishment surpassed fear in accordance with De Lombaert et al. (2017). Vigorous mouth-object contacts could be detected either on day 2 during training or on day 3 during NOR tests (Fig. 3). Aluminum foil balls and plastic pearls provoked similar exploratory behavior no significant differences in the numbers of mouth-object contacts on day 2 during training (Fig. 4). When either floating plastic pearl or floating aluminum foil ball was used as novel object, significantly higher number of mouth-object contacts were recorded on day 3 during NOR test (Fig. 5). Therefore, according to the results described above, the type of floating object had no significant effect on the intensity of exploratory behavior so that any innate preference can be ruled out in each case. Since zebrafish in their natural habitat are often feeding close to water surface, consequently smaller floating and non-stationary objects as potential preys attract exploring fish even more than submerged stationary underwater objects. Such stationary objects may provoke neophobia in zebrafish depending on shape and size as reported by May et al. (2016) who suspected that fish perceived larger objects as predators. Zebrafish tested in this pilot study showed neophilia in each experiment according to the results described above, whereas smaller floating objects, close to prey size, were used as novel objects. Sudden changes in refraction inducing bright and twinkling light signals together with intense red (as suggested by Spence and Smith 2008; Avdesh et al. 2012) and other attractive colors (mimicked hereby using floating crumpled aluminum foil balls, and pink plastic hollow pearls) are observed by fish feeding close to surface and presumably considered as insects or similar floating preys. We suggest further extensive research in order to determine the optimal shapes, size, surface properties and colors of floating novel objects to be used in NOR studies on zebrafish.

## Conclusion

Floating objects such as crumpled aluminum foil balls and pink plastic hollow pearls are suitable for novel object recognition tests (NOR) when small groups of adult zebrafish are used as experimental animals. Adult zebrafish of both sexes were found to be capable of distinguishing between the different colors and surface consistencies of certain floating objects. Floating novel objects can influence exploratory behavior of adult zebrafish significantly.
